# Supplementary material for: Prognostic Impact of Dihydropyrimidine Dehydrogenase Germline Variants in Unresectable Non-Small Cell Lung Cancer Patients Treated with Platin-Based Chemotherapy
Source: Int J Mol Sci. 2023 Jun 7;24(12):9843. doi: 10.3390/ijms24129843 (PMC10298094; doi:10.3390/ijms24129843)
Supplement: Supplementary file 1 [file ijms-24-09843-s001.zip › ijms-2399119-supplementary.pdf]

SNP	Gene	Transcript ID
rs4244285	*CYP2C19*	NM_000769.2
rs4986893	*CYP2C19*	NM_000769.2
rs12248560	*CYP2C19*	NM_000769.2
rs28399504	*CYP2C19*	NM_000769.2
rs56337013	*CYP2C19*	NM_000769.2
rs72552267	*CYP2C19*	NM_000769.2
rs72558186	*CYP2C19*	NM_000769.2
rs41291556	*CYP2C19*	NM_000769.2
rs17884712	*CYP2C19*	NM_000769.2
rs6413438	*CYP2C19*	NM_000769.2
rs192154563	*CYP2C19*	NM_000769.2
rs1564657013	*CYP2C19*	NM_000769.2
rs118203759	*CYP2C19*	NM_000769.2
rs375781227*	*CYP2C19*	NM_000769.2
rs140278421	*CYP2C19*	NM_000769.2
rs118203757	*CYP2C19*	NM_000769.2
rs12769205	*CYP2C19*	NM_000769.2
rs4986910	*CYP3A4*	NM_017460.5
rs35599367	*CYP3A4*	NM_017460.5
rs776746	*CYP3A5*	NM_000777.4
rs10264272	*CYP3A5*	NM_000777.4
rs41303343	*CYP3A5*	NM_000777.4
rs1799853	*CYP2C9*	NM_000771.3
rs1057910	*CYP2C9*	NM_000771.3
rs28371686	*CYP2C9*	NM_000771.3
rs9332131	*CYP2C9*	NM_000771.3
rs7900194	*CYP2C9*	NM_000771.3
rs28371685	*CYP2C9*	NM_000771.3
rs9332239	*CYP2C9*	NM_000771.3
rs72558187	*CYP2C9*	NM_000771.3
rs72558190	*CYP2C9*	NM_000771.3
rs11572103	*CYP2C8*	NM_000770.3
rs10509681	*CYP2C8*	NM_000770.3
rs1058930	*CYP2C8*	NM_000770.3
rs1800460	*TPMT*	NM_000367.3
rs1142345	*TPMT*	NM_000367.3
rs1800462	*TPMT*	NM_000367.3
rs1800584	*TPMT*	NM_000367.3
rs116855232	*NUDT15*	NM_018283.3
rs3918290	*DPYD*	NM_000110.3
rs55886062	*DPYD*	NM_000110.3
rs67376798	*DPYD*	NM_000110.3
rs75017182	*DPYD*	NM_000110.3

rs72549309	*DPYD*	NM_000110.3
rs1801266	*DPYD*	NM_000110.3
rs1801268	*DPYD*	NM_000110.3
rs78060119	*DPYD*	NM_000110.3
rs56038477	*DPYD*	NM_000110.3
rs115232898	*DPYD*	NM_000110.3
rs887829	*UGT1A1*	NM_000463.2
rs4148323	*UGT1A1*	NM_000463.2
rs35350960	*UGT1A1*	NM_000463.2
rs1801133	*MTHFR*	NM_005957.4
rs987237	*TFAP2B*	NM_003221.3
rs1867785	*EPAS1*	NM_001430.4
rs1048719	*GM2A*	NM_000405.4
rs11465996	*LY96*	NM_015364.5

Table S1: SNPs included in our custom pharmacogenetic assay.
